# Prevalence and Associated Factors of Smoking Among Final Year Medical Students: A Multicentric Survey From Pakistan

**DOI:** 10.7759/cureus.701

**Published:** 2016-07-18

**Authors:** Mohammad U Khubaib, Zuhaib Y Shahid, Sameed K Lodhi, Hamza Malik, Mohsin M Jan

**Affiliations:** 1 Department of Rehabilitation Medicine, CMH Lahore Medical College and Institute of Dentistry; 2 House Officer, Jinnah Hospital Lahore (JHL)/Allama Iqbal Medical College (AIMC), Lahore, Pakistan; 3 House Officer, King Edward Medical University(KEMU)/Mayo Hospital, Lahore, Pakistan; 4 Surgery, CMH Lahore Medical College and Institute of Dentistry; 5 House Job, CMH Lahore Medical College and Institute of Dentistry

**Keywords:** cigarette, tobacco, epidemiology, final year mbbs, doctors, physician, lahore, medical school, demography, developing countries

## Abstract

Introduction

Smoking is the leading cause of lung cancer around the world. In a developing country like Pakistan with low levels of literacy and general awareness about adverse effects of smoking, doctors play a pivotal role in educating the masses about its harmful consequences and providing support for smoking cessation. However, their efficacy is affected if they smoke themselves, and oftentimes the habits cultivated during educational recourse are carried into the professional careers. The aim of this study was to document the prevalence of smoking among final year medical students of Lahore, Pakistan, and the factors associated with it.

Methodology

Study approval was obtained from Combined Military Hospital (CMH) Lahore Medical College, Ethics Review Committee. A cross-sectional survey was carried out in four medical colleges and hospitals of Lahore, Pakistan. A questionnaire consisting of 14 questions related to basic demographics and smoking was used after being pilot tested on 20 students of CMH. The overall response rate was 74.89%. Data was collected from 337 respondents, of which 38 forms were discarded and 299 forms were analyzed by SPSS V21.

Results

Among the 299 respondents, there were 128 males (42.81%) and 171 females (57.19%) with 32 (10.70%) smokers. Male students reported smoking (n = 27, 21.09%) more than their female counterparts (n = 5, 0.02%). The mean age of participants was 23.01 years. Students having an active smoker at home had statistically significant positive correlations with current smoking status and the number of cigarettes smoked per day. Students with household smoking contacts were also more likely to smoke if they belonged to the male gender.

Conclusion

Prevalence of smoking in medical students is lower than in the general population but still considerable in the male students. There is a need to target this particular population with interactive counseling sessions, education campaigns, and anti-smoking rules to decrease smoking among them and through them in the society.

## Introduction

Smoking has many adverse effects on health and is the leading cause of preventable morbidity and mortality all around the globe [1]. It is associated with a variety of diseases, including but not limited to chronic obstructive pulmonary disease, lung cancer, cardiovascular disease, stroke, cataracts, infertility, birth defects, miscarriage, and rheumatoid arthritis in both active and passive smokers [2-4]. Smoking kills approximately six million people annually [5]. However, smoking cessation reduces the risk of major diseases dramatically and, in some cases, to the same level as that of non-smokers [6]. It is more common in developing countries like Pakistan, which has the highest prevalence of male smokers (about 45%) in South Asia. Interestingly enough, smoking cigarettes decreases with education, with only 18% of men with higher education being smokers [7].

Bachelor of Medicine, Bachelor of Surgery (MBBS) students spend five years in medical schools learning about disease, pathology, prevention, and treatment and are well aware of smoking hazards and health risks. With a literacy rate of only 55% [8], the majority of Pakistan’s population is uneducated and oblivious to the deleterious effects of smoking. In Pakistan, doctors are revered and their advice is placed in high esteem. Therefore, they can serve as ambassadors of smoking prevention and cessation and reduce the knowledge gap regarding hazards.

Final year medical students are less than a year away from becoming practicing doctors, and therefore, their attitude towards smoking dictates how successful they will be in convincing their smoking patients to quit.

This study aims to determine the prevalence of smoking in final year medical students in Lahore, Pakistan, to identify factors responsible for smoking among them, and to ascertain their attitudes towards smoking cessation.

## Materials and methods

Study approval was obtained from the Ethics Review Committee, Combined Military Hospital (CMH), Lahore Medical College. A cross-sectional study design was used. The data collection form had 14 questions in total. The first three questions addressed basic demographics. The remaining 11 questions were about smoking and included questions about current and past smoking status, the presence and number of household contacts who smoked, age when smoking started, reasons for starting smoking, number of smokes per day, cessation attempts and their forms, reasons for trying to quit, quit success, and intention to quit if currently smoking. The questionnaire was reviewed by a faculty member of the Department of Community Medicine, CMH Lahore Medical College.

This questionnaire was then pilot tested on 20 students of CMH Lahore Medical College, yielding an average time required to fill the questionnaire of about five minutes. Nobody had any confusion or trouble understanding it. It was then distributed to 450 final year MBBS students in four different medical colleges in Lahore, including Allama Iqbal Medical College (AIMC), Shaikh Khalifa Bin Zayed Al-Nahyan Medical and Dental College (SZMDC), Lahore Medical and Dental College (LMDC) and Shalamar Medical and Dental College (SMDC). Of these, 337 forms were returned (response rate = 74.89%). Data was collected by the authors or their representatives who were briefed in advance and informed consent was verbally obtained. Thirty-eight of the 337 forms were discarded due to faulty or incomplete entries, and data from 299 forms was entered.

Data was analyzed in SPSS v. 21. Chi-square test was used to analyze correlations between categorical variables, including smokers at home, the age group at which smoking was started, the number of cigarettes smoked, and gender. T-Test was used to analyze age of the medical students and its relationship with the number of cigarettes smoked. Continuous variables were represented by the respective value and standard error of mean (SEM). A P-value of less than 0.05 was considered statistically significant.

## Results

There were 299 respondents in total. Out of these, 128 (42.8%) were males and 171 (57.2%) were females. The number of current smokers was 32. This was 10.7 % of our study population. A noticeably higher proportion of males (n = 27, 21.09%) reported current smoking as compared to females (n = 5, 0.02%). The mean age of the students was 23.01 ± 0.057. The age at which students smoked the most was 23. Some of the significant categorical variables are listed in Table 1.

**Table 1:** Categorical Variables of Smokers

Variable	Frequency
Participants with smokers at home	57
Smoking in the past	36
Quit attempt successful	8
Intention to quit	29

The correlation between students who currently smoked and had someone smoking at home was statistically significant (P-value = 0.005). The student was more likely to smoke if there was someone at home smoking as well, with a higher chance of the smoking student being male (P-value = 0.053). Students who had never smoked a cigarette in their life did not have any smokers in their homes (P-value < 0.0001).

If the student did not have anyone at home smoking cigarettes, he/she was more likely to start smoking at a later age. This correlation proved to be statistically significant as well according to our study (P-value < 0.0001) (Table 2).

**Table 2:** Age of Starting Smoking

Smokers at Home	Yes	No
< 15 years	6	3
15-19 years	9	6
> 20 years	0	16

The main reason for starting to smoke in both genders was recreational. Males, however, were the only ones smoking for relaxation as well.

The students were divided into age groups, and the correlation with the number of cigarettes smoked was determined as seen in Table 3.

**Table 3:** Number of Cigarettes Smoked in a Day

Age when smoking was started	< 15 years	15-19 years	> 20 years
1-5 cigarettes	3	10	12
6-19 cigarettes	2	4	4
> 20 cigarettes	4	1	0

The Spearman correlation coefficient was -0.370, which showed a weak relationship, but a statistically significant one as the P-value was 0.019. This showed that the greater the age of starting smoking, the greater the likelihood to smoke fewer cigarettes as compared to those who started at an early age.

## Discussion

The aim of this study was to estimate the prevalence of smoking among medical students and the factors associated with it. Our study identified several important aspects, including a relatively low prevalence of smoking among medical students as compared to general population. The majority of males and females started smoking for recreational purposes; however, some of the male students reported that smoking helped them relax and relieve stress, which might explain why most of the current smokers began to smoke after 20 years of age when many had already started medical education. Interestingly, most of the smokers (n = 29) reported an intention to quit. Moreover, it was found that there was a noticeable difference in smoking prevalence between male and female genders, with female smoking being almost a negligible phenomenon among medical students. While this represents a satisfactory trend as far as female medical students are concerned, it actually distorts the overall smoking figures as the reality is that most of the male medical students continue on to practice medicine while many female students end up not practicing medicine after graduation [9] and the prevalence of smoking in male medical students is almost double that of the overall medical students smoking percentage. The study also indicated that having household contacts who smoked was a significant predictor of smoking, especially among male smokers. Another important finding was that participants were more likely to start smoking at earlier ages and consume a higher number of cigarettes per day if they had household smoking contacts.

Similar smoking prevalences have been highlighted in surveys from individual medical colleges of Pakistan in the past as well. From Lahore, CMH Lahore Medical College and King Edward Medical University Lahore had 10.6% and 13.45% students who smoked, respectively [10-11]. From Karachi, Hamdard University had 14.5% smokers while Agha Khan University Karachi had 14.4% smokers [12-13].

Prevalence of smoking is increasing exponentially, especially in the developing countries. By the year 2030, approximately 70% of deaths attributable to smoking worldwide are expected to occur in developing countries [14]. Pakistan has the highest consumption of tobacco in South Asia and 45% of the male population smokes, which reduces to 18% in men with higher education [7]. The State Bank statistical bulletin reports that Pakistanis smoked 64.48 billion cigarettes in the fiscal year 2014 [15]. Smoking is responsible for many health hazards in Pakistan. It is reported that lung cancer in Pakistan is caused directly by tobacco in 90% of cases and claims the lives of 100,000 people every year [16]. Furthermore, people who smoke are 7 to 16 times more likely to indulge in illicit drug abuse, such as marijuana and heroin, leading to further problems related to individual and social health [17].

It is the need of the hour that doctors as well as medical students give health education to the community and promote anti-smoking policies. Simple advice from doctors and other healthcare professionals has been found to significantly increase smoking cessation rates [18]. Moreover, research shows that smoking physicians are less likely than non-smoking ones to advise cessation [19]. Therefore, it is vital that medical students, being future physicians, practice healthy behavior themselves to be more effective in their counseling.

Government and medical colleges should invest in awareness sessions with doctors and medical students to emphasize their important role in smoking eradication from the community. Laws should be imposed to curb smoking in public settings, and educational institutions, in particular, should be declared no-smoking zones. This should have a beneficial effect in counteracting some of the most common reasons for starting smoking, such as 'recreational' or 'peer pressure'. Medical institutions should also make on-campus specialized counselors available for 'stressed-out' medical students to educate them about the healthy activities to relieve stress, instead of making matters worse through smoking. Last, but not the least, early on in their medical education, students with smokers at home should be identified and counseled to advise and educate members of their household about the risks associated with smoking and the benefits of smoking cessation. This would deal with one of the biggest determinants of smoking status in medical students.

As with all research, our study also has some limitations. Firstly, our study only included final year medical students and, thus, excluded practicing doctors as well as other allied health professionals. Secondly, as it was a cross-sectional study, causality could not be tested. Lastly, the questionnaire was self-administered so some students may not have provided accurate responses.

## Conclusions

While smoking in medical students is much lower than its high prevalence in the general population of Pakistan, a considerable percentage of male final-year students smoke. Considering the huge disease burden that smoking contributes to in the country, it is important that final-year medical students, who are the doctors of tomorrow, stop smoking so that they can advise their future patients to do so as well. For this, the government and the medical institutions need to strictly control smoking among doctors and medical students to improve and increase the health of the society.
